# A Case Study of the Relationship between Basic Psychological Needs and General Health among Old People

**Published:** 2017-12

**Authors:** Azam BAZOOBAND, Abdolvahab BAGHBANIAN, Ghazal TORKFAR

**Affiliations:** 1. Dept. of Management, Babol Branch, Payame Noor University, Babol, Iran; 2. Faculty of Health Sciences, The University of Sydney, Sydney, Australia; 3. Menzies Center for Health Policy, Sydney School of Public Health, The University of Sydney, Sydney, Australia

## Dear Editor-in-Chief

Mental health provides intellectual and communication skills and can increase the human’s emotions, resilience and self-esteem or the successful performance of mental functions and as a result doing productive activities, having good relations with others, the ability to adapt to changes and deal effectively with negative life events are the consequences of mental health (1). Regarding the increase of aged population around the world, mental health issues among elderly has attracted researches attitude. Seniors are vulnerable to mental health disorders during their ageing process (2, 3).

Human beings have physical, social and psychological needs that provide their satisfaction of life; so the researches in the area of needs have focused on the development of basic psychological needs in recent years raised for the first time in 2000 with title of Self-determination theory (4). Overall satisfying basic psychological needs, lead to increase mental health and psychological well-being and if there is an obstacle, the mental health and psychological well-being is reduced in an individual (5, 6).

This study was a descriptive survey conducted among the elderly living in Jam, Boushehr Province, southern Iran. The sample size was selected by using Cochran formula and included 92 people; Questionnaires were distributed among them with simple random sampling method.

In this study which has been done in 2016–2017, the questionnaire of basic psychological needs (Basic Needs Satisfaction) and GHQ (General Health Questioner) was used. Informed consent was taken from participants prior to commencement of the study.

The most respondents were in the age group of 56 to 66 yr age group (with a 52.4%) and then 67 to 77 yr (with a value of 28.6%) and only 4.8% of respondents were 55 yr or less. 97.6% of respondents were women and 2.4 percent of respondents were men. General health was measured by 28 items in four dimensions. The mean score of general health was 50.30 that is less than 70, the average score. The score was lower than the average indicator of general health and higher scores indicate disorder. Therefore, the elderly have favorable general health. In addition, basic psychological needs were measured by 21 items in three dimensions; the mean score of respondents from the basic psychological needs was 77.81 that are greater than the average score of 63. A higher score indicates better psychological well-being. Therefore, the basic psychological needs of the elderly were satisfied.

There was a significant negative correlation between psychological well-being and general health (sig= 0/001, r= −0/66). The lowest correlation between the subscales of psychological well-being and mental health needs of autonomy (r= −0/42) and the highest correlation coefficient related to general health (r=−0/ 77), which suggests a strong relationship between competency and general health. The general health can provides necessary factors for satisfying the basic needs.

The results of simple regression analysis showed that psychological well-being was a significant predictor for general health. This variable explains 43% of the variation in the dependent variable. In addition, only the competency subscale was able to predict the general health. The competency totally explained 59% of variance in general health (R2= 59%).

With regard to the relationship between basic psychological needs and general health, the researcher suggests officials and those involved in organizations associated with the elderly specially their families in addition to the economic support of them, to pay especial attention to mental empowerment approaches, with an emphasis on satisfying basic psychological needs such as the need for autonomy and competency.
